# Bis(tetra­ethyl­ammonium) oxalate dihydrate

**DOI:** 10.1107/S160053681203022X

**Published:** 2012-07-07

**Authors:** Timothy J. McNeese, Robert D. Pike

**Affiliations:** a Department of Chemistry, Loyola University Maryland, 4501 North Charles Street, Baltimore, MD 21210-2699, USA; b Department of Chemistry, College of William and Mary, PO Box 8795, Williamsburg, VA 23187-8795, USA

## Abstract

The title compound, 2C_8_H_20_N^+^·C_2_O_4_^2−^·2H_2_O, synthesized by neutralizing H_2_C_2_O_4_·2H_2_O with (C_2_H_5_)_4_NOH in a 1:2 molar ratio, is a deliquescent solid. The oxalate ion is nonplanar, with a dihedral angle between carboxyl­ate groups of 64.37 (2)°. O—H⋯O hydrogen bonds of moderate strength link the O atoms of the water mol­ecules and the oxalate ions into rings parallel to the *c* axis. The rings exhibit the graph-set motif *R*_4_^4^(12). In addition, there are weak C—H⋯O inter­actions in the crystal structure.

## Related literature

For related compounds containing planar and nonplanar oxalate ions, see: Beagley & Small (1964 ▶); Robertson (1965 ▶); Jeffrey & Parry (1954 ▶). For general syntheses of tetra­alkyl­ammonium salts and their uses, see: Barthel & Kunz (1988 ▶); Heck (1982 ▶); Markowitz (1957 ▶); McNeese *et al.* (1984 ▶); Starks (1971 ▶). For uses of [(C_2_H_5_)_4_N)_2_(C_2_O_4_)]·2H_2_O, see: Darensbourg *et al.* (1992 ▶); Demadis & Coucouvanis (1995 ▶); Diop *et al.* (1997 ▶); Engels *et al.* (1983 ▶). For classification of the graph-set motifs, see: Etter *et al.* (1990 ▶). For classification of the hydrogen bonds, see: Gilli & Gilli (2009 ▶). Oxalate was confirmed by the blue ring resorcinol test (Chernoff, 1920 ▶).
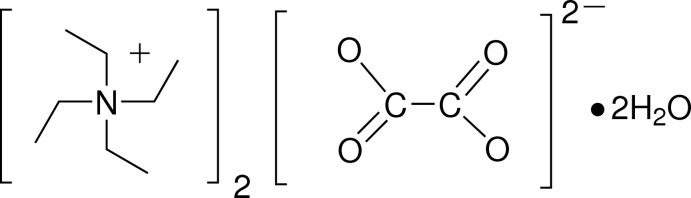


## Experimental

### 

#### Crystal data

2C_8_H_20_N^+^·C_2_O_4_^2−^·2H_2_O
*M* *_r_* = 384.55
Orthorhombic, 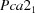

*a* = 19.9302 (4) Å
*b* = 7.6627 (1) Å
*c* = 14.3253 (3) Å
*V* = 2187.75 (7) Å^3^
*Z* = 4
Cu *K*α radiation
μ = 0.70 mm^−1^
*T* = 100 K
0.32 × 0.25 × 0.05 mm

#### Data collection

Bruker SMART APEXII CCD diffractometer
Absorption correction: multi-scan (*SADABS*; Sheldrick, 2004 ▶) *T*_min_ = 0.808, *T*_max_ = 0.966
17374 measured reflections
2026 independent reflections
1953 reflections with *I* > 2σ(*I*)
*R*_int_ = 0.067

#### Refinement

*R*[*F* ^2^ > 2σ(*F* ^2^)] = 0.031
*wR*(*F* ^2^) = 0.081
*S* = 1.07
2026 reflections
256 parameters
1 restraint
H atoms treated by a mixture of independent and constrained refinement
Δρ_max_ = 0.20 e Å^−3^
Δρ_min_ = −0.16 e Å^−3^

### 

Data collection: *APEX2* (Bruker, 2004 ▶); cell refinement: *SAINT-Plus* (Bruker, 2004 ▶); data reduction: *SAINT-Plus*; program(s) used to solve structure: *SHELXS97* (Sheldrick, 2008 ▶); program(s) used to refine structure: *SHELXLE* (Hübschle *et al.*, 2011 ▶); molecular graphics: *ORTEP-3* (Farrugia, 1997 ▶) and *Mercury* (Macrae *et al.*, 2006 ▶); software used to prepare material for publication: *SHELXL97* (Sheldrick, 2008 ▶).

## Supplementary Material

Crystal structure: contains datablock(s) I, global. DOI: 10.1107/S160053681203022X/fb2256sup1.cif

Structure factors: contains datablock(s) I. DOI: 10.1107/S160053681203022X/fb2256Isup2.hkl

Supplementary material file. DOI: 10.1107/S160053681203022X/fb2256Isup3.cml

Additional supplementary materials:  crystallographic information; 3D view; checkCIF report

## Figures and Tables

**Table 1:** Hydrogen-bond geometry (Å, °)

*D*—H⋯*A*	*D*—H	H⋯*A*	*D*⋯*A*	*D*—H⋯*A*
O6—H1*W*⋯O1^i^	0.82 (4)	1.96 (4)	2.749 (2)	161 (3)
O6—H2*W*⋯O3^ii^	0.87 (4)	2.00 (4)	2.842 (2)	162 (3)
O5—H3*W*⋯O2^iii^	0.86 (4)	1.88 (4)	2.720 (2)	166 (4)
O5—H4*W*⋯O4^iv^	0.85 (4)	1.89 (4)	2.732 (2)	167 (3)
C3—H3*B*⋯O2^i^	0.99	2.40	3.300 (3)	151
C5—H5*A*⋯O1	0.99	2.43	3.324 (3)	149
C5—H5*B*⋯O4^iii^	0.99	2.34	3.270 (3)	157
C7—H7*A*⋯O3^ii^	0.99	2.44	3.332 (3)	150
C8—H8*B*⋯O4^iii^	0.98	2.57	3.497 (3)	158
C10—H10*A*⋯O2^i^	0.98	2.50	3.450 (3)	162
C11—H11*B*⋯O5^v^	0.99	2.41	3.384 (3)	167
C13—H13*A*⋯O1^vi^	0.99	2.37	3.334 (3)	165
C15—H15*B*⋯O4^vi^	0.99	2.51	3.166 (3)	124
C16—H16*C*⋯O6^vii^	0.98	2.59	3.559 (3)	170
C18—H18*A*⋯O3^i^	0.98	2.59	3.296 (2)	129

Symmetry codes: (i) 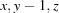; (ii) 
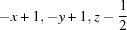
; (iii) 
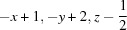
; (iv) 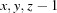; (v) 
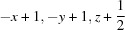
; (vi) 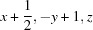; (vii) 
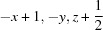
.

**Fig. 1.**
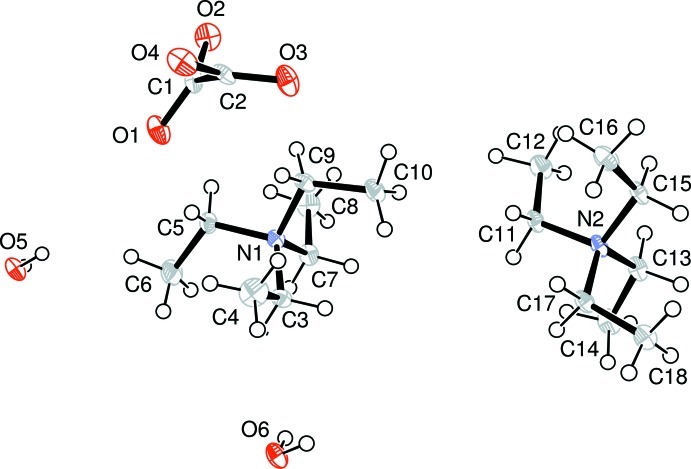
Ellipsoid plot of the title molecules. Displacement ellipsoids are drawn at the 50% probability level.

**Fig. 2.**
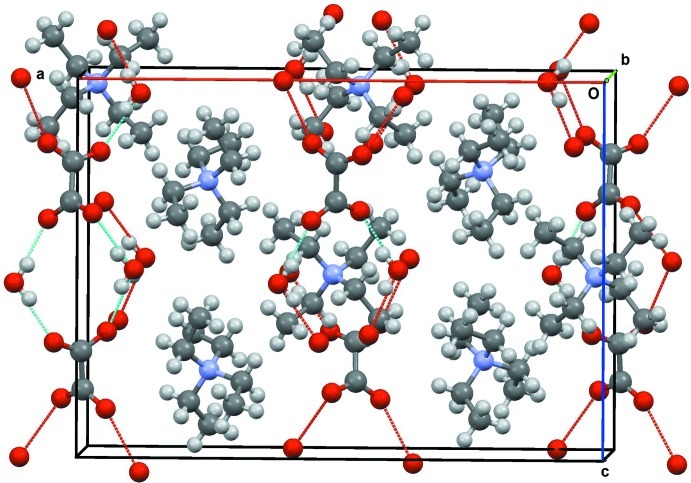
Mercury (Macrae et al., 2006) packing diagram of the title compound viewed down the b axis.

## supplementary crystallographic information

### Comment

Tetraalkylammonium salts are important compounds as phase-transfer catalysts (Starks, 1971), as electrolytes in electrochemical studies (Barthel & Kunz, 1988), in organic Heck-type synthetic reactions (Heck, 1982), and in preparing and isolating polynuclear organometallic complexes (McNeese *et al.*, 1984). A procedure for synthesizing quaternary ammonium salts involves neutralization of a tetraalkylammonium hydroxide with the acid of the desired anion (Markowitz, 1957).

The title compound, (Et_4_N)_2_C_2_O_4_.2H_2_O, synthesized by reaction of Et_4_NOH and H_2_C_2_O_4_.2H_2_O in a 2:1 mole ratio, is deliquescent and rapidly absorbs moisture from air. Previously prepared and used *in situ* or as a noncrystalline solid, it has been employed as a reductant in aprotic electrochemical cells (Engels *et al.*, 1983) and in the synthesis of oxalate-containing organometallic complexes (Diop *et al.*, 1997; Demadis & Coucouvanis, 1995; Darensbourg *et al.*, 1992).

Oxalate bond distances (C—C and C—O) and angles (O—C—O and O—C—C) are comparable to other reported oxalate salts containing NH_4_^+^ and alkali metal ions. The oxalate ion is planar in Li_2_C_2_O_4_ (Beagley & Small, 1964) and Na_2_C_2_O_4_ (Jeffrey & Parry, 1954), but the dihedral angle between planes of symmetrical carboxylate groups of the oxalate fragment is 26.6° in (NH_4_)_2_C_2_O_4_.H_2_O (Robertson, 1965). The directional character of the hydrogen bonding pattern in the monohydrate is believed responsible for the observed non-planar stereochemistry of the oxalate ion. A dihedral angle of 64.37 (2)° is present in the title compound (Fig. 1). The non-planarity of the oxalate ion is maintained by moderate hydrogen bonds (Table 1) that link the oxygen atoms of the oxalate ion and the water molecules into a ring motif *R*^4^_4_(12) (Etter *et al.*, 1990). (For the classification of the hydrogen bonds, see Gilli & Gilli, 2009). In addition, there are also present weak C-H···O interactions in the structure (Tab. 1). Fig. 2 illustrates the packing diagram for the structure of the title compound.

### Experimental

An aqueous 35 weight percent solution of Et_4_NOH (6.69 g, 15.9 mmol OH^-^) was added by syringe to a 50-ml Schlenk tube containing a 20-ml CH_3_CN solution of H_2_C_2_O_4_.2H_2_O (1.00 g, 7.93 mmol). The colorless solution was stirred under argon for 15 min followed by solvent removal under reduced pressure. Hot tetrahydrofuran (25 ml) was added to the greasy solid, the mixture was stirred for 10 min, and the solution was decanted from the product. The deliquescent white solid was dried under vacuum and crystallized from CH_3_CN/ THF. Yield: 1.98 g (65%). IR (υ(CO), CH_3_CN) 1565(*s*) cm^-1^. Oxalate was confirmed by the blue ring resorcinol test (Chernoff, 1920). The analyzed crystal was rapidly transferred from vacuum to a 100 K stream of dry air for X-ray analysis.

### Refinement

Data were refined against F^2^. Because of the relatively high *R*_int_ the determination of the absolute structure turned out to be meaningless and therefore 1509 Friedel pairs have been merged. All the hydrogen atoms appeared in the difference electron density map, nevertheless, those pertinent to the methyl and the methylene carbons were situated into the idealized positions and refined in the riding atom formalism. The positional parameters of the water hydrogens were refined freely. The applied constraints: C_methyl_-H_methyl_=0.98, C_methylene_-H_methylene_=0.99 Å. *U*_iso_(H_methylene_) = 1.2*U*_eq_(C_methylene_), *U*_iso_(H_methyl_) = 1.5*U*_eq_(C_methyl_), *U*_iso_(H_water_) = 1.5*U*_eq_(O_water_).

### Crystal data

**Table:**

2C_8_H_20_N^+^·C_2_O_4_^2^^−^·2H_2_O	*F*(000) = 856
*M**_r_* = 384.55	*D*_x_ = 1.168 Mg m^−^^3^
Orthorhombic, *P**c**a*2_1_	Cu *K*α radiation, λ = 1.54178 Å
Hall symbol: P 2c -2ac	Cell parameters from 6094 reflections
*a* = 19.9302 (4) Å	θ = 3.8–68.4°
*b* = 7.6627 (1) Å	µ = 0.70 mm^−^^1^
*c* = 14.3253 (3) Å	*T* = 100 K
*V* = 2187.75 (7) Å^3^	Plate, colorless
*Z* = 4	0.32 × 0.25 × 0.05 mm

### Data collection

**Table:**

Bruker SMART APEXII CCD diffractometer	2026 independent reflections
Radiation source: fine-focus sealed tube	1953 reflections with *I* > 2σ(*I*)
Graphite monochromator	*R*_int_ = 0.067
ω and ψ scans	θ_max_ = 67.0°, θ_min_ = 4.4°
Absorption correction: multi-scan (*SADABS*; Sheldrick, 2004)	*h* = −23→23
*T*_min_ = 0.808, *T*_max_ = 0.966	*k* = −8→9
17374 measured reflections	*l* = −16→16

### Refinement

**Table:**

Refinement on *F*^2^	Secondary atom site location: difference Fourier map
Least-squares matrix: full	Hydrogen site location: difference Fourier map
*R*[*F*^2^ > 2σ(*F*^2^)] = 0.031	H atoms treated by a mixture of independent and constrained refinement
*wR*(*F*^2^) = 0.081	*w* = 1/[σ^2^(*F*_o_^2^) + (0.0468*P*)^2^ + 0.3359*P*] where *P* = (*F*_o_^2^ + 2*F*_c_^2^)/3
*S* = 1.07	(Δ/σ)_max_ < 0.001
2026 reflections	Δρ_max_ = 0.20 e Å^−^^3^
256 parameters	Δρ_min_ = −0.16 e Å^−^^3^
1 restraint	Extinction correction: *SHELXLE* (Hübschle *et al.*, 2011), Fc^*^=kFc[1+0.001xFc^2^λ^3^/sin(2θ)]^-1/4^
152 constraints	Extinction coefficient: 0.0018 (3)
Primary atom site location: structure-invariant direct methods	

### Special details

**Table:**

Geometry. All e.s.d.'s (except the e.s.d. in the dihedral angle between two l.s. planes) are estimated using the full covariance matrix. The cell e.s.d.'s are taken into account individually in the estimation of e.s.d.'s in distances, angles and torsion angles; correlations between e.s.d.'s in cell parameters are only used when they are defined by crystal symmetry. An approximate (isotropic) treatment of cell e.s.d.'s is used for estimating e.s.d.'s involving l.s. planes.
Refinement. Refinement of *F*^2^ against ALL reflections. The weighted *R*-factor *wR* and goodness of fit *S* are based on *F*^2^, conventional *R*-factors *R* are based on *F*, with *F* set to zero for negative *F*^2^. The threshold expression of *F*^2^ > σ(*F*^2^) is used only for calculating *R*-factors(gt) *etc*. and is not relevant to the choice of reflections for refinement. *R*-factors based on *F*^2^ are statistically about twice as large as those based on *F*, and *R*- factors based on ALL data will be even larger.

### Fractional atomic coordinates and isotropic or equivalent isotropic displacement parameters (Å^2^)

**Table:**

	*x*	*y*	*z*	*U*_iso_*/*U*_eq_	
O1	0.44416 (8)	0.9370 (2)	0.66940 (11)	0.0260 (4)	
O2	0.54192 (8)	1.0741 (2)	0.69474 (11)	0.0245 (4)	
O3	0.53292 (7)	0.8233 (2)	0.84977 (11)	0.0263 (4)	
O4	0.44453 (8)	0.9997 (2)	0.87126 (11)	0.0254 (4)	
C1	0.49202 (10)	0.9852 (3)	0.71976 (14)	0.0162 (4)	
C2	0.48956 (10)	0.9310 (3)	0.82269 (14)	0.0167 (4)	
N1	0.52560 (8)	0.5362 (2)	0.52871 (12)	0.0155 (4)	
C3	0.48056 (10)	0.3892 (3)	0.56294 (15)	0.0191 (4)	
H3A	0.4527	0.3484	0.5100	0.023*	
H3B	0.5092	0.2903	0.5826	0.023*	
C4	0.43481 (12)	0.4368 (3)	0.64282 (19)	0.0321 (6)	
H4A	0.4618	0.4682	0.6975	0.048*	
H4B	0.4061	0.3369	0.6581	0.048*	
H4C	0.4067	0.5363	0.6248	0.048*	
C5	0.48413 (10)	0.6851 (3)	0.48890 (15)	0.0194 (4)	
H5A	0.4561	0.7342	0.5396	0.023*	
H5B	0.5151	0.7782	0.4678	0.023*	
C6	0.43882 (13)	0.6362 (3)	0.40826 (19)	0.0331 (6)	
H6A	0.4200	0.7423	0.3804	0.050*	
H6B	0.4023	0.5620	0.4310	0.050*	
H6C	0.4648	0.5726	0.3612	0.050*	
C7	0.57042 (10)	0.4560 (3)	0.45509 (15)	0.0191 (4)	
H7A	0.5418	0.4061	0.4053	0.023*	
H7B	0.5958	0.3587	0.4837	0.023*	
C8	0.61999 (11)	0.5824 (3)	0.41095 (17)	0.0267 (5)	
H8A	0.6519	0.6226	0.4584	0.040*	
H8B	0.5956	0.6828	0.3854	0.040*	
H8C	0.6444	0.5233	0.3606	0.040*	
C9	0.56591 (10)	0.6129 (3)	0.60804 (14)	0.0180 (4)	
H9A	0.5347	0.6644	0.6542	0.022*	
H9B	0.5943	0.7084	0.5833	0.022*	
C10	0.61047 (11)	0.4815 (3)	0.65726 (16)	0.0231 (5)	
H10A	0.5836	0.3801	0.6759	0.035*	
H10B	0.6303	0.5355	0.7128	0.035*	
H10C	0.6463	0.4440	0.6149	0.035*	
N2	0.74829 (8)	0.0031 (2)	0.76685 (12)	0.0164 (4)	
C11	0.72670 (10)	0.1585 (3)	0.70941 (15)	0.0189 (4)	
H11A	0.6954	0.2301	0.7470	0.023*	
H11B	0.7019	0.1160	0.6540	0.023*	
C12	0.78410 (11)	0.2737 (3)	0.67665 (17)	0.0257 (5)	
H12A	0.8076	0.3218	0.7310	0.039*	
H12B	0.7663	0.3694	0.6386	0.039*	
H12C	0.8154	0.2043	0.6392	0.039*	
C13	0.79440 (10)	−0.1148 (3)	0.71088 (15)	0.0195 (4)	
H13A	0.8343	−0.0467	0.6918	0.023*	
H13B	0.8100	−0.2106	0.7518	0.023*	
C14	0.76283 (11)	−0.1938 (3)	0.62449 (16)	0.0243 (5)	
H14A	0.7287	−0.2792	0.6430	0.036*	
H14B	0.7975	−0.2519	0.5873	0.036*	
H14C	0.7418	−0.1014	0.5873	0.036*	
C15	0.78792 (10)	0.0602 (3)	0.85239 (15)	0.0204 (4)	
H15A	0.8001	−0.0449	0.8888	0.024*	
H15B	0.8302	0.1154	0.8312	0.024*	
C16	0.75169 (11)	0.1864 (3)	0.91622 (15)	0.0247 (5)	
H16A	0.7430	0.2955	0.8827	0.037*	
H16B	0.7796	0.2103	0.9711	0.037*	
H16C	0.7090	0.1349	0.9362	0.037*	
C17	0.68437 (10)	−0.0925 (3)	0.79498 (14)	0.0177 (4)	
H17A	0.6562	−0.0122	0.8324	0.021*	
H17B	0.6590	−0.1220	0.7377	0.021*	
C18	0.69534 (10)	−0.2576 (3)	0.85037 (15)	0.0213 (4)	
H18A	0.6519	−0.3112	0.8646	0.032*	
H18B	0.7187	−0.2296	0.9087	0.032*	
H18C	0.7226	−0.3391	0.8138	0.032*	
O5	0.38115 (7)	0.9648 (2)	0.03930 (11)	0.0216 (3)	
H1W	0.4148 (16)	0.023 (4)	0.547 (3)	0.032*	
H2W	0.4216 (17)	0.071 (4)	0.461 (3)	0.032*	
O6	0.39111 (7)	0.0424 (2)	0.50150 (12)	0.0235 (3)	
H3W	0.4112 (17)	0.951 (4)	0.082 (3)	0.035*	
H4W	0.4061 (16)	0.968 (4)	−0.009 (3)	0.035*	

### Atomic displacement parameters (Å^2^)

**Table:**

	*U*^11^	*U*^22^	*U*^33^	*U*^12^	*U*^13^	*U*^23^
O1	0.0228 (8)	0.0430 (9)	0.0122 (8)	0.0059 (6)	−0.0052 (6)	−0.0044 (7)
O2	0.0276 (8)	0.0317 (8)	0.0143 (8)	−0.0002 (6)	0.0056 (6)	0.0056 (6)
O3	0.0217 (7)	0.0387 (9)	0.0185 (8)	0.0006 (6)	−0.0022 (6)	0.0122 (7)
O4	0.0247 (8)	0.0400 (9)	0.0114 (7)	−0.0035 (7)	0.0055 (6)	−0.0047 (6)
C1	0.0175 (10)	0.0226 (10)	0.0085 (9)	0.0064 (7)	0.0016 (8)	−0.0021 (8)
C2	0.0128 (9)	0.0265 (10)	0.0109 (10)	−0.0050 (7)	−0.0016 (8)	0.0008 (8)
N1	0.0153 (8)	0.0194 (8)	0.0120 (8)	−0.0007 (7)	−0.0004 (7)	−0.0002 (6)
C3	0.0176 (9)	0.0211 (10)	0.0187 (10)	−0.0045 (8)	−0.0009 (8)	0.0010 (8)
C4	0.0275 (12)	0.0354 (13)	0.0334 (14)	−0.0027 (10)	0.0150 (11)	0.0028 (10)
C5	0.0199 (9)	0.0203 (9)	0.0181 (10)	0.0036 (7)	−0.0028 (8)	0.0008 (8)
C6	0.0365 (13)	0.0322 (12)	0.0306 (13)	0.0062 (10)	−0.0179 (11)	−0.0034 (10)
C7	0.0188 (10)	0.0249 (10)	0.0134 (10)	0.0039 (8)	0.0026 (8)	−0.0021 (8)
C8	0.0243 (11)	0.0342 (11)	0.0216 (11)	0.0024 (9)	0.0090 (9)	0.0016 (9)
C9	0.0172 (9)	0.0242 (10)	0.0125 (10)	−0.0029 (8)	−0.0015 (8)	−0.0014 (8)
C10	0.0226 (11)	0.0291 (11)	0.0176 (11)	−0.0012 (8)	−0.0068 (9)	0.0012 (9)
N2	0.0121 (7)	0.0252 (9)	0.0119 (8)	−0.0003 (7)	−0.0011 (7)	0.0019 (7)
C11	0.0161 (9)	0.0250 (10)	0.0156 (9)	0.0006 (8)	−0.0026 (8)	0.0053 (9)
C12	0.0217 (10)	0.0316 (11)	0.0239 (11)	−0.0041 (9)	−0.0012 (9)	0.0084 (9)
C13	0.0141 (9)	0.0296 (10)	0.0149 (10)	0.0031 (8)	0.0032 (8)	0.0019 (9)
C14	0.0239 (11)	0.0331 (11)	0.0159 (10)	0.0019 (8)	0.0035 (8)	−0.0017 (9)
C15	0.0171 (9)	0.0300 (11)	0.0140 (10)	0.0002 (8)	−0.0055 (8)	0.0014 (8)
C16	0.0249 (10)	0.0346 (11)	0.0146 (10)	−0.0017 (9)	−0.0041 (8)	−0.0032 (9)
C17	0.0120 (9)	0.0279 (11)	0.0132 (10)	−0.0013 (8)	0.0007 (7)	0.0008 (8)
C18	0.0216 (10)	0.0296 (11)	0.0128 (10)	−0.0006 (8)	0.0014 (8)	0.0014 (8)
O5	0.0169 (7)	0.0371 (8)	0.0109 (7)	−0.0003 (6)	0.0004 (7)	0.0001 (6)
O6	0.0195 (8)	0.0351 (8)	0.0159 (8)	0.0004 (6)	−0.0043 (7)	0.0009 (7)

### Geometric parameters (Å, º)

**Table:**

O1—C1	1.252 (3)	N2—C11	1.510 (3)
O2—C1	1.258 (3)	N2—C13	1.518 (3)
O3—C2	1.256 (3)	N2—C15	1.522 (3)
O4—C2	1.252 (3)	N2—C17	1.524 (2)
C1—C2	1.533 (3)	C11—C12	1.520 (3)
N1—C9	1.511 (2)	C11—H11A	0.9900
N1—C7	1.513 (2)	C11—H11B	0.9900
N1—C5	1.520 (3)	C12—H12A	0.9800
N1—C3	1.522 (3)	C12—H12B	0.9800
C3—C4	1.508 (3)	C12—H12C	0.9800
C3—H3A	0.9900	C13—C14	1.514 (3)
C3—H3B	0.9900	C13—H13A	0.9900
C4—H4A	0.9800	C13—H13B	0.9900
C4—H4B	0.9800	C14—H14A	0.9800
C4—H4C	0.9800	C14—H14B	0.9800
C5—C6	1.514 (3)	C14—H14C	0.9800
C5—H5A	0.9900	C15—C16	1.514 (3)
C5—H5B	0.9900	C15—H15A	0.9900
C6—H6A	0.9800	C15—H15B	0.9900
C6—H6B	0.9800	C16—H16A	0.9800
C6—H6C	0.9800	C16—H16B	0.9800
C7—C8	1.521 (3)	C16—H16C	0.9800
C7—H7A	0.9900	C17—C18	1.509 (3)
C7—H7B	0.9900	C17—H17A	0.9900
C8—H8A	0.9800	C17—H17B	0.9900
C8—H8B	0.9800	C18—H18A	0.9800
C8—H8C	0.9800	C18—H18B	0.9800
C9—C10	1.517 (3)	C18—H18C	0.9800
C9—H9A	0.9900	O5—H3W	0.87 (4)
C9—H9B	0.9900	O5—H4W	0.86 (4)
C10—H10A	0.9800	O6—H1W	0.82 (4)
C10—H10B	0.9800	O6—H2W	0.87 (4)
C10—H10C	0.9800		
			
O1—C1—O2	126.7 (2)	H10A—C10—H10C	109.5
O1—C1—C2	116.77 (19)	H10B—C10—H10C	109.5
O2—C1—C2	116.50 (18)	C11—N2—C13	110.73 (16)
O4—C2—O3	126.7 (2)	C11—N2—C15	111.11 (16)
O4—C2—C1	116.34 (18)	C13—N2—C15	106.39 (15)
O3—C2—C1	116.95 (18)	C11—N2—C17	106.56 (15)
C9—N1—C7	111.62 (15)	C13—N2—C17	111.08 (16)
C9—N1—C5	106.21 (16)	C15—N2—C17	111.05 (16)
C7—N1—C5	111.38 (16)	N2—C11—C12	114.34 (16)
C9—N1—C3	111.07 (16)	N2—C11—H11A	108.7
C7—N1—C3	105.78 (16)	C12—C11—H11A	108.7
C5—N1—C3	110.86 (15)	N2—C11—H11B	108.7
C4—C3—N1	114.95 (18)	C12—C11—H11B	108.7
C4—C3—H3A	108.5	H11A—C11—H11B	107.6
N1—C3—H3A	108.5	C11—C12—H12A	109.5
C4—C3—H3B	108.5	C11—C12—H12B	109.5
N1—C3—H3B	108.5	H12A—C12—H12B	109.5
H3A—C3—H3B	107.5	C11—C12—H12C	109.5
C3—C4—H4A	109.5	H12A—C12—H12C	109.5
C3—C4—H4B	109.5	H12B—C12—H12C	109.5
H4A—C4—H4B	109.5	C14—C13—N2	114.72 (16)
C3—C4—H4C	109.5	C14—C13—H13A	108.6
H4A—C4—H4C	109.5	N2—C13—H13A	108.6
H4B—C4—H4C	109.5	C14—C13—H13B	108.6
C6—C5—N1	115.14 (17)	N2—C13—H13B	108.6
C6—C5—H5A	108.5	H13A—C13—H13B	107.6
N1—C5—H5A	108.5	C13—C14—H14A	109.5
C6—C5—H5B	108.5	C13—C14—H14B	109.5
N1—C5—H5B	108.5	H14A—C14—H14B	109.5
H5A—C5—H5B	107.5	C13—C14—H14C	109.5
C5—C6—H6A	109.5	H14A—C14—H14C	109.5
C5—C6—H6B	109.5	H14B—C14—H14C	109.5
H6A—C6—H6B	109.5	C16—C15—N2	114.97 (16)
C5—C6—H6C	109.5	C16—C15—H15A	108.5
H6A—C6—H6C	109.5	N2—C15—H15A	108.5
H6B—C6—H6C	109.5	C16—C15—H15B	108.5
N1—C7—C8	114.48 (17)	N2—C15—H15B	108.5
N1—C7—H7A	108.6	H15A—C15—H15B	107.5
C8—C7—H7A	108.6	C15—C16—H16A	109.5
N1—C7—H7B	108.6	C15—C16—H16B	109.5
C8—C7—H7B	108.6	H16A—C16—H16B	109.5
H7A—C7—H7B	107.6	C15—C16—H16C	109.5
C7—C8—H8A	109.5	H16A—C16—H16C	109.5
C7—C8—H8B	109.5	H16B—C16—H16C	109.5
H8A—C8—H8B	109.5	C18—C17—N2	114.90 (16)
C7—C8—H8C	109.5	C18—C17—H17A	108.5
H8A—C8—H8C	109.5	N2—C17—H17A	108.5
H8B—C8—H8C	109.5	C18—C17—H17B	108.5
N1—C9—C10	113.75 (17)	N2—C17—H17B	108.5
N1—C9—H9A	108.8	H17A—C17—H17B	107.5
C10—C9—H9A	108.8	C17—C18—H18A	109.5
N1—C9—H9B	108.8	C17—C18—H18B	109.5
C10—C9—H9B	108.8	H18A—C18—H18B	109.5
H9A—C9—H9B	107.7	C17—C18—H18C	109.5
C9—C10—H10A	109.5	H18A—C18—H18C	109.5
C9—C10—H10B	109.5	H18B—C18—H18C	109.5
H10A—C10—H10B	109.5	H3W—O5—H4W	100 (3)
C9—C10—H10C	109.5	H1W—O6—H2W	100 (3)
			
O1—C1—C2—O4	−67.8 (3)	C5—N1—C9—C10	179.55 (17)
O2—C1—C2—O4	111.9 (2)	C3—N1—C9—C10	58.9 (2)
O1—C1—C2—O3	112.5 (2)	C13—N2—C11—C12	−60.6 (2)
O2—C1—C2—O3	−67.8 (3)	C15—N2—C11—C12	57.4 (2)
C9—N1—C3—C4	54.2 (2)	C17—N2—C11—C12	178.52 (18)
C7—N1—C3—C4	175.46 (18)	C11—N2—C13—C14	−62.2 (2)
C5—N1—C3—C4	−63.7 (2)	C15—N2—C13—C14	176.99 (17)
C9—N1—C5—C6	−179.36 (19)	C17—N2—C13—C14	56.0 (2)
C7—N1—C5—C6	58.9 (2)	C11—N2—C15—C16	56.9 (2)
C3—N1—C5—C6	−58.6 (2)	C13—N2—C15—C16	177.47 (17)
C9—N1—C7—C8	−59.2 (2)	C17—N2—C15—C16	−61.6 (2)
C5—N1—C7—C8	59.3 (2)	C11—N2—C17—C18	177.74 (17)
C3—N1—C7—C8	179.88 (18)	C13—N2—C17—C18	57.1 (2)
C7—N1—C9—C10	−58.9 (2)	C15—N2—C17—C18	−61.1 (2)

### Hydrogen-bond geometry (Å, º)

**Table:**

*D*—H···*A*	*D*—H	H···*A*	*D*···*A*	*D*—H···*A*
O6—H1*W*···O1^i^	0.82 (4)	1.96 (4)	2.749 (2)	161 (3)
O6—H2*W*···O3^ii^	0.87 (4)	2.00 (4)	2.842 (2)	162 (3)
O5—H3*W*···O2^iii^	0.86 (4)	1.88 (4)	2.720 (2)	166 (4)
O5—H4*W*···O4^iv^	0.85 (4)	1.89 (4)	2.732 (2)	167 (3)
C3—H3*B*···O2^i^	0.99	2.40	3.300 (3)	151
C5—H5*A*···O1	0.99	2.43	3.324 (3)	149
C5—H5*B*···O4^iii^	0.99	2.34	3.270 (3)	157
C7—H7*A*···O3^ii^	0.99	2.44	3.332 (3)	150
C8—H8*B*···O4^iii^	0.98	2.57	3.497 (3)	158
C10—H10*A*···O2^i^	0.98	2.50	3.450 (3)	162
C11—H11*B*···O5^v^	0.99	2.41	3.384 (3)	167
C13—H13*A*···O1^vi^	0.99	2.37	3.334 (3)	165
C15—H15*B*···O4^vi^	0.99	2.51	3.166 (3)	124
C16—H16*C*···O6^vii^	0.98	2.59	3.559 (3)	170
C18—H18*A*···O3^i^	0.98	2.59	3.296 (2)	129

Symmetry codes: (i) *x*, *y*−1, *z*; (ii) −*x*+1, −*y*+1, *z*−1/2; (iii) −*x*+1, −*y*+2, *z*−1/2; (iv) *x*, *y*, *z*−1; (v) −*x*+1, −*y*+1, *z*+1/2; (vi) *x*+1/2, −*y*+1, *z*; (vii) −*x*+1, −*y*, *z*+1/2.
